# Functional differentiation of 3-ketosteroid Δ^1^-dehydrogenase isozymes in *Rhodococcus ruber* strain Chol-4

**DOI:** 10.1186/s12934-017-0657-1

**Published:** 2017-03-14

**Authors:** Govinda Guevara, Laura Fernández de las Heras, Julián Perera, Juana María Navarro Llorens

**Affiliations:** 1 Department of Biochemistry and Molecular Biology I, Universidad Complutense de Madrid, 28040 Madrid, Spain; 2 Faculty of Science and Engineering, Microbial Physiology-Gron Inst Biomolecular Sciences & Biotechnology, Nijenborgh 7, 9747 AG Groningen, The Netherlands

**Keywords:** *Rhodococcus ruber*, 3-Ketosteroid-∆^1^-dehydrogenase, Promoters, Expression, Steroids

## Abstract

**Background:**

The *Rhodococcus ruber* strain Chol-4 genome contains at least three putative 3-ketosteroid Δ^1^-dehydrogenase ORFs (*kstD1*, *kstD2* and *kstD3*) that code for flavoenzymes involved in the steroid ring degradation. The aim of this work is the functional characterization of these enzymes prior to the developing of different biotechnological applications.

**Results:**

The three *R. ruber* KstD enzymes have different substrate profiles. KstD1 shows preference for 9OHAD and testosterone, followed by progesterone, deoxy corticosterone AD and, finally, 4-BNC, corticosterone and 19OHAD. KstD2 shows maximum preference for progesterone followed by 5α-Tes, DOC, AD testosterone, 4-BNC and lastly 19OHAD, corticosterone and 9OHAD. KstD3 preference is for saturated steroid substrates (5α-Tes) followed by progesterone and DOC. A preliminary attempt to model the catalytic pocket of the KstD proteins revealed some structural differences probably related to their catalytic differences. The expression of *kstD* genes has been studied by RT-PCR and RT-qPCR. All the *kstD* genes are transcribed under all the conditions assayed, although an additional induction in cholesterol and AD could be observed for *kstD1* and in cholesterol for *kstD3*. Co-transcription of some correlative genes could be stated. The transcription initiation signals have been searched, both in silico and in vivo. Putative promoters in the intergenic regions upstream the *kstD1, kstD2* and *kstD3* genes were identified and probed in an apramycin-promoter-test vector, leading to the functional evidence of those *R. ruber kstD* promoters.

**Conclusions:**

At least three putative 3-ketosteroid Δ^1^-dehydrogenase ORFs (*kstD1*, *kstD2* and *kstD3*) have been identified and functionally confirmed in *R. ruber* strain Chol-4. KstD1 and KstD2 display a wide range of substrate preferences regarding to well-known intermediaries of the cholesterol degradation pathway (9OHAD and AD) and other steroid compounds. KstD3 shows a narrower substrate range with a preference for saturated substrates. KstDs differences in their catalytic properties was somehow related to structural differences revealed by a preliminary structural modelling. Transcription of *R. ruber kstD* genes is driven from specific promoters. The three genes are constitutively transcribed, although an additional induction is observed in *kstD1* and *kstD3*. These enzymes have a wide versatility and allow a fine tuning-up of the KstD cellular activity.

**Electronic supplementary material:**

The online version of this article (doi:10.1186/s12934-017-0657-1) contains supplementary material, which is available to authorized users.

## Background

Rhodococci are aerobic Gram-positive soil bacteria belonging to the Actinomycetes group. They show a broad catabolic diversity over different substrates, from pollutants to many aromatic compounds, including steroids and sterols [1–3]. Steroids are a source of contamination of soil and waters and their presence has been detected even in drinking water, threatening many ways of life and public health [4–6]. Rhodococci can be useful in this biodegradation field due to their metabolic versatility and steroids degradation capability. On the other hand *Rhodococcus* spp. are potential biotechnological tools [3, 7] as they can provide with key enzymes essential for certain reactions that yield industrial needed intermediaries such as 4-androstene-3,17-dione (AD) and 1,4-androstadiene-3,17-dione (ADD) [8].

But before exploiting all the advantages the different rhodococci offer, it is essential to know how these bacteria degrade steroids and which enzymes are involved in this process.

Steroids are molecules with a carbon skeleton of 4 fused rings (A to D) and a side chain up to 10 carbons. During the last years, the increasing number of studies concerning steroid degradation, and more concretely the degradation of cholesterol in bacteria, have clarified some of the catabolic steps (e.g. initiation of the ring degradation by either a NAD^+^-dependent 3β-hydroxysteroid dehydrogenase or a cholesterol oxidase) although other steps still remain unclear (e.g. the processing of the C and D rings of the steroid structure or the relative order in which the different steps of the degradation of ring and chain occurs) [3, 9–12].

In the general scheme of steroid degradation, there are two key enzymes that initiate the opening of the steroid ring: the 3-ketosteroid-∆^1^-dehydrogenase [4-ene-3-oxosteroid: (acceptor)-1-ene-oxoreductase; EC 1.3.99.4)], also known as KstD and the 3-ketosteroid 9α-hydroxylase [Androsta-1,4-diene-3,17-dione; EC 1.14.13.142], also known as Ksh [13]. KstD is a flavoenzyme involved in the ∆^1^-dehydrogenation of the steroid molecule leading to the initiation of the breakdown of the steroid nucleus by introducing a double bond into the A-ring of 3-ketosteroids [14, 15]. This flavoprotein converts 4-ene-3-oxosteroids (e.g. AD) to 1,4-diene-3-oxosteroids (e.g. ADD) by trans-axial elimination of the C-1(α) and C-2(β) hydrogen atoms [16]. KstD homologs have been identified in 100 different bacterial species (78 actinobacteria, 20 proteobacteria and 2 firmicutes) and at least in one fungus, *Aspergillus fumigatus* CICC 40167 [17, 18]. Most of these KstD-containing bacteria occur in soil, marine or river sediments and are also able to degrade polycyclic aromatic hydrocarbons [19]. Phylogenetic analysis leads to classify the KstD-like enzymes in at least 4 different groups, in which KstD1, KstD2, KstD3 of *Rhodococcus erythropolis* SQ1 are representatives of three of them [20]. The crystal structure of the enzyme KstD1 of *R. erythropolis* SQ1 has been elucidated [21] confirming the presence of the two domains previously described, namely a N-terminal flavin adenine dinucleotide (FAD) binding motif and a substrate-binding domain [14, 20, 22, 23].

The substrate range of different KstD proteins has been studied in *R. erythropolis* SQ1, being 3-ketosteroids with a saturated A-ring (e.g. 5α-androstane-3,17-dione and 5α-testosterone) the preferred substrates for KstD3 and (9α-hydroxy-)4-androstene-3,17-dione the favourite one for both KstD1 and KstD2 [20]. It should be mentioned that, apart from their role in steroids degradation, KstD proteins could have specific roles depending of their origin; for instance, the KstD of *A. fumigatus* CICC 40167 is involved in fusidane antibiotic biosynthesis [17].

We have previously reported the occurrence of three KstD enzymes in *R. ruber* (NCBI::AFH57399 for KstD1; NCBI::AFH57395 for KstD2 and NCBI::ACS73883 for KstD3) [24]. Growth experiments with single, double or triple *kstD* mutants proved that KstD2 is a key enzyme in the transformation of both AD to ADD and 9α-hydroxy-4-androstene-3,17-dione (9OHAD) to 9α-hydroxy-1,4-androstadiene-3,17-dione (9OHADD) while both KstD2 and KstD3 are involved in the cholesterol catabolism in *R. ruber*. On the other hand, the role of KstD1 on the steroids catabolism remains unclear as *kstD1* mutation did not affect growing of this strain in steroids [24]. In this study, we cloned the three *kstD* ORFs and heterologously expressed them in *R. erythropolis* CECT3014, in order to initiate the biochemical characterization of the encoded enzymes, as the basis for further studies on their applications. The results revealed that KstD3 uses more actively substrates with a saturated ring in contrast to KstD1 and KstD2. Additionally, we located and functionally defined the promoters of the three *kstD* ORFS in order to provide a basis for future research on the regulation of these genes.

## Methods

### Bacterial strains, plasmids and growth conditions

*Rhodococcus ruber* strain Chol-4 (CECT 7469; DSM 45280) was isolated from a sewage sludge sample [25]. This strain was routinely grown in Luria-Bertani (LB) or minimal medium (M457 of the DSMZ, Braunschweig, Germany) containing the desired carbon and energy source under aerobic conditions at 30 °C in a rotary shaker (250 rpm) for 1–3 days. For the steroids growth experiments, a LB pre-grown culture was washed two times with minimal medium prior to inoculation. Cholesterol or AD (Sigma), were added directly to the minimal medium culture for growing and/or induction at 0.6 and 0.44 g/L, respectively. *Escherichia coli* strains were grown in LB broth at 37 °C, 250 rpm. For the promoter growth experiments, cells were plated in minimal medium M457 plates containing the desired carbon source and incubated at 30 °C for 3 days. Cholesterol and AD were previously dissolved in methyl-β-cyclodextrin (CD) [26] and prepared as described [27]. Plasmids and bacterial strains used are listed in Additional file 1. Competent cells of *E. coli* DH5αF’ and BL21 (DE3) were prepared and transformed by standard protocols [28].

### Cloning of *kstD1*, *kstD2* and *kstD3* of *R. ruber* strain Chol-4 and heterologous expression in *Rhodococcus erythropolis* CECT3014 cells

Chromosomal DNA extraction of *R. ruber* grown in a LB agar plate was performed using the hexadecyltrimethylammonium bromide (CTAB) procedure [29] with the following modifications. Bacterial cells were collected, suspended in 400 µL Tris–EDTA buffer (10 mM Tris/HCl, pH 8.1 mM EDTA) and incubated at 80 °C for 20 min. Afterwards, a lysozyme treatment (50 µL of 10 mg/mL stock) was carried out at 37 °C for 1–12 h, and then 75 µL of SDS containing proteinase K (70 µL SDS 10% wt/vol plus 5 µL proteinase K 10 mg/mL) was added and incubated for 10 min at 65 °C. Proteins were precipitated with 100 µL of 5 M NaOH and 100 µL CTAB (0.1 g/mL suspended in 0.7 M NaOH) for 10 min at 65 °C. DNA was purified by extraction with chloroform-isoamyl alcohol (24:1) and phenol-chloroform-isoamyl alcohol (25:24:1) and precipitated with 0.6 vol of isopropanol at room temperature for 30 min. After centrifugation, DNA was washed with 70% ethanol and suspended in distilled water.

The *kstD* ORFs were previously identified using the Bioedit program [25] and they were PCR amplified, from start to stop codon, using primers from Additional file 2. PCR was performed under standard conditions using High Fidelity PCR Enzyme Mix (Fermentas) with a specific high GC buffer (Roche) at 30 cycles of 1 min at 95 °C, 1 min at the desired Tm and 0.5–3 min at 72 °C (unless stated otherwise).

*Nde*I-*Bam*HI (*kstD3*), *Nde*I-*Bgl*II (*kstD2* and *kstD1*) restricted PCR products were first cloned into pGEM-T Easy Vector (Promega) and then moved to the shuttle vector pTip-QC1 [30]. Expression plasmids either with or without a *kstD* ORF were used to electroporate *Rhodococcus erythropolis* CECT3014. Selection was made in LB with 34 µg/mL Cm. Cultures of *R. erythropolis* harbouring expression plasmids (pTip-KsTD1, pTip-KstD2, pTip-KstD3 or pTip-QC1) were grown (30 °C, 200 rpm) in 50 mL LB broth supplemented with Cm until an OD_600nm_ of 0.6–0.8. After that, expression was induced by adding 1 µg/mL thiostrepton (Sigma) to the culture. Cells were kept growing for 24 h more and they were collected at 5000 rpm for 10 min, washed twice in 50 mM phosphate buffer pH 7.0, concentrated to 5 mL and sonicated. The resulting cell extracts were used for analysis of KstD activity with a range of steroid substrates. Total protein content was measured by Bradford assay [31]. Samples were analysed by PAGE-SDS in 12.5% wt/vol gels and using 10 µg of total protein per lane.

### KstD enzymatic assay

The kinetic of the enzymatic extracts was determined as previously stated [20, 32]. The cell-free extracts were incubated with AD (Sigma), 9OHAD (Organon Biosciences), 17β-hydroxy-5α-androstan-3-one or 5α-Tes, DOC (deoxycorticosterone), testosterone, corticosterone, 19OHAD, progesterone (Sigma), 4-BNC (4-pregnen-3-one-20β-carboxylic acid) (Steraloids), 5β-androstane-3,17-dione (Steraloids), 4-cholestene-3-one (Sigma) or 5α-cholestan-3-one (Acros Organics). Structures of steroids used in this study are shown in Additional file 3. Enzyme activities were measured spectrophotometrically at 30 °C using 2,6-dichlorophenol-indophenol (DCPIP, Sigma) as an artificial electron acceptor. The reaction mixture (1 mL) consisted of 50 mM Tris (pH 7), 80 μM DCPIP, cell-free extract and 200 μM steroid in ethanol (methanol in the case of 5α-cholestane-3-one). Four replicates were analysed. Activities are expressed as mean values ± SD in units per milligram of protein; one unit is defined as the amount of enzyme which causes the reduction of 1 µmol of DCPIP/min (ε_600_ = 21 mM^−1^ cm^−1^) after taking into account the value of the activity of control (cells harbouring the empty pTip-QC1 vector) for a certain steroid. Total protein concentration (mg/mL) was measured by Bradford assay [31]. The kinetics of the KstD enzymes were determined by incubating the cell-free extracts with varying concentrations of steroid substrates. The kinetics parameters were analysed by nonlinear regression curve fitting of the data to the Michaelis- Menten equation using Hyper32 1.0 software (Informer Technologies, http://hyper32.software.informer.com/).

### Expression analysis by RT-PCR and RT-qPCR

RNA samples for RT-PCR experiments were obtained from mid-log exponential phase cultures (OD_600nm_ 0.7–0.8). Total RNA was prepared with the RNeasy Mini Kit (Qiagen) following the manufacturer’s indications with the following modification: 50 mg of acid-washed glass beads (150 μm diameter) were added in the first step and each sample was shaken at maximum speed in a Bullet Blender for 5 min. The cell debris was removed by centrifugation. The supernatant was subjected to the RNeasy Mini Kit (Qiagen) protocol. The total RNA obtained (0.5–1 μg) was treated once with 5 U of Turbo DNase RNase-Free (Ambion) in a 700 μL volume for 2 h at 37 °C. RNA samples were extracted with 1 volume of acid phenol (Sigma), vigorously shaken and incubated at room temperature for 15 min. After 15 min centrifugation, the upper phase was precipitated by addition of 0.12 volumes of 5 M NH_4_Ac, 0.02 volumes of glycogen (5 mg/mL) and 1 volume of isopropanol, washed twice in 70% ethanol and dissolved in water. Samples were treated with DNase until no DNA was detected by PCR to avoid DNA contamination. The RNA concentration was then evaluated using a NanoDrop Spectrophotometer ND-1000.

For the RT-PCR the cDNA was synthesized using SuperScrip III Reverse Transcriptase (Invitrogen) following the manufacturer’s indications. cDNA was used as template (25 ng) for PCR reactions (20 μL final volume). Controls without reverse transcriptase (RT-) were used to detect any contamination of undigested DNA in the RNA preparations. PCR products were analysed in 0.8% agarose gels.

To quantify the expression of the three KstD genes, a RT-qPCR analysis was performed using RNA from wild-type strains cultured in M457 minimal medium containing the desired carbon and energy source (2 g/L sodium acetate, 0.44 g/L AD or 0.6 g/L cholesterol). The RNA quality was assessed by using Bioanalyzer 2100 (Agilent). cDNA was synthetized using 1 μg of RNA with the high capacity RNA to cDNA Kit (Applied Biosystems). The RT-qPCR analysis of cDNA was performed on Applied Biosystems QuantStudio 12K Flex Real-Time PCR Systems. The reaction conditions were 10 min at 95 °C followed by 40 cycles of 15 s at 95 °C and 1 min at 60 °C for extension. The temperature of the melting curve was from 60 to 95 °C. The FAD-binding dehydrogenase D092_14375 gene was used as an internal control to normalize messenger RNA levels. All reactions were performed in triplicate. The RT-qPCR experiment and the analysis of the relative fold difference of each gene using the 2^−∆∆Ct^ algorithm was performed in the Genomic unit of Universidad Complutense de Madrid.

The sodium acetate grown culture was used as the reference medium. Therefore, the relative expression indicates how many times the expression level of a certain gene is detected respect to the levels detected when growing on sodium acetate.

### In silico analyses

DNASTAR (Lasergene) programs were used to analyse sequences and to design primers. The *R. ruber* strain Chol-4 genomic DNA has been previously sequenced [33]. BioEdit program was used to perform local-blast alignments within the genome data (NCIB::ANGC01000000). Putative signal peptides were predicted by SignalP 4.1 server using a model trained on Gram-positive bacteria [34]. Sigma 70 putative promoters predictions were performed using the BPROM server, a bacterial sigma 70 promoter recognition program with about 80% accuracy and specificity [35], the Neural Network Promoter Prediction (NNPP) based on prokaryotes [36] in all cases with a score value of ≥80%, or the webserver PePPER for prediction of prokaryote promoter elements and regulons [37].

For the protein modelling we employed different software. I-Tasser (http://zhanglab.ccmb.med.umich.edu/I-TASSER) was used for an approach to protein structure and function prediction [38] and PredictProtein (www.predictprotein.org/home) was used for the secondary structure, solvent accessibility and transmembrane helix prediction [39]. COBALT was used as a multiple sequence alignment tool to find similarities among the catalytic residues (www.ncbi.nlm.nih.gov/tools/cobalt/re_cobalt.cgi) [40] and PyMOL (www.pymol.org/) as a molecular visualization system (PyMOL Molecular Graphics System, Version 0.99, Schrödinger).

### Promoter cloning and characterization

The *Nhe*I-*Pci*I 0.4 Kb multiple cloning site (mcs) from pSEVA351 [41, 42] was cloned into pNV119 vector [43], from now on named as pNVS (Additional file 4).

The putative *kstD* promoter sequences were amplified by PCR, from the end of the upstream flanking gene to the end of the first six amino acid codifying sequence. The *Xba*I*, Pst*I-flanked *kstD* promoter regions (*kstD1p, kstD2p* and *kstD3p*) and the *Kpn*I, *Pst*I-flanked *kstD3* ^*b*^ *p* minimal promoter were cloned into pNVS. The resulting vectors were designated pNVSP1, pNVSP2, pNVSP3 and pNVSP3^b^, respectively.

Apramycin resistance gene (Am^r^) was amplified by PCR from plasmid pIJ773 [44], from start to stop codon. The *Nru*I/*Hin*dII digested Am^r^ fragment was cloned in each one of the previous *kstD* constructions. The resulting plasmids were checked by sequencing (Secugen) and named pNVSP1-A, pNVSP2-A, pNVSP3-A and pNVSP3^b^-A, respectively.

All the primers used are listed in Additional file 2. PCR was performed under standard conditions using High Fidelity PCR (Roche) with glycerol 5% and a basic program unless stated otherwise.

As a control, a plasmid without any promoter but carrying the Am^r^ gene (pNVSA) was made by digesting pNVSP1-A with *Nru*I-*Xba*I; the resulting 4.2 Kb fragment was cut from a 1% agarose gel and purified with GENECLEAN Turbo Kit. Blunt ends suitable for ligation with T4 DNA ligase (Takara) were generated using the End repair kit (DNA terminator, Lucigen). The final ligated product was used to transform *E. coli* DH5αF′. Deletion was checked by sequencing (Secugen).

Every one of the plasmid set was introduced in *R. ruber* strain Chol-4 by electroporation (200 or 400 µL cells with 1 µg DNA at 400 Ω, 25 mA, 2.5 µF; 10–11 ms), the resulting cells were suspended in 800 µL of LB and kept for 6 min at 46 °C, and then for 5 h at 30 °C without shaking. Finally, they were plated on LB Agar with 200 μg/mL kanamycin and kept at 30 °C. To verify the presence of plasmids, two colonies of each plate were picked and grown in 3 mL of LB-200 μg/mL kanamycin. Plasmid were extracted using the method described in Hopwood et al. [45] and used to transform *E. coli* strain DH5αF’ [28]. Plasmids obtained from *E. coli* colonies grown at 37 °C in 50 μg/mL kanamycin were verified by sequencing (Secugen). Finally, those colonies of *R. ruber* strain Chol-4 harbouring the right recombinant plasmids were picked and grown in agar minimal media at 30 °C with different carbon source with or without apramycin (300 μg/mL) or kanamycin (200 μg/mL).

To define the transcriptional start sites (TSS) of *kstD* promoters, a transcription start point protocol (ARF-TSS) [46] was used on *R. ruber* cells with the following modifications.

RNA from *R. ruber* cells growing in different carbons sources cultures was isolated. The culture media were: LB for *kstD1* TSS, minimal medium supplemented with AD for *kstD2* TSS and minimal medium supplemented with cholesterol for *kstD3* TSS. Total RNA was isolated as described previously [47]. It was qualified by electrophoresis and quantified by Nanodrop 1000 (NanoDrop Technologies). 20 µg of RNA were reverse transcribed with a gene specific phosphorylated 5′-end primer (R1 for each *kstD*) using SuperScrip III Reverse Transcriptase from Invitrogen. As a result, cDNA fragments with the TSS as 3′-end were generated and purified. After removing of RNA with 0.5 µg/µL RNase A (37 °C for 30 min), cDNA was purified using the GENECLEAN turbo Kit (MPI) and final products were treated with T4 RNA ligase from Fermentas (10U, 37 °C for 30 min). This T4 RNA ligase catalyses the ATP-dependent intra- and intermolecular formation of phosphodiester bonds between 5′-phosphate and 3′-hydroxyl termini of oligonucleotides, single-stranded RNA and DNA. It was used in this study to circularize the cDNA. The circularized cDNAs were then used as template for PCR amplification with Expand High Fidelity (Roche) using R2 and F3 primers specific for each *kstD* ORF. PCR products were purified using the GENECLEAN turbo Kit and sequenced (Secugen). The nucleotide upstream the 5′-end of the R1 primer is the transcription initiation site.

## Results and discussion

As we have published earlier, the *R. ruber* strain Chol-4 genome contains three putative 3-ketosteroid Δ^1^-dehydrogenase ORFs (*kstD1*, *kstD2* and *kstD3*) that code for flavoenzymes involved in the steroid ring degradation [24]. Growth experiments with *kstD* mutants proved that KstD2 is the main enzyme involved in the transformation of AD to ADD. *R. ruber kstD2* mutants accumulate 9OHAD from AD due to the action of a 3-ketosteroid-9α-hydroxylase (KshAB). On the other hand, only the strains lacking both KstD2 and KstD3 were unable to grow in minimal medium with cholesterol as the only carbon source. In order to know more about these *R. ruber* enzymes, we have performed transcriptional studies and followed their heterologous expression in *R. erythropolis* to detect their activities on a set of different substrates.

### In silico analyses of *kstD* promoter regions of *R. ruber* strain Chol-4

**Fig. 1:**
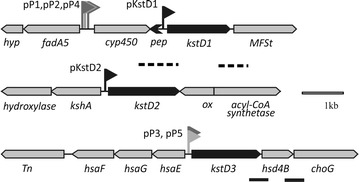
Schematic representation of *R. ruber* strain Chol-4 DNA *kstD* regions. Putative promoters (pP) predicted using the BPROM (pP1: TTCCTT^−35^..TGCTTGAAT^−10^), PePPER (pP2: TTGAATGCTTTTAGAACGTGTTCCACATCgcgaC^+1^ and pP3: TGGACTCACCGCGCCATCATTCTATAACgtgtT^+1^) or NNPP programs (pP4: GGTTGTCGTGGCGGACAAGGTGTGGTCCGAATGATCGGGAC^+1^TTGGCGATT and pP5: GAAGGGATGGACTCACCGCGCCATCATTC^+1^TATAACGTG) are shown in grey flags. TSS derived promoters (pKstD1, pKstD2) appear in black flags. Positive (*solid line*) and negative (*dotted line*) results of the amplification of co-transcribed products are depicted. *hyp* hypothetical protein, *fadA5* acetyl-CoA acyltransferase, *cyp450* cytochrome P450, *pep* hypothetical peptide, *MFSt* major facilitator superfamily transporter, *kshA* 3-ketosteroid 9α-hydroxylase, *ox* oxidoreductase, *Tn transposase*, *hsaF* 4-hydroxy-2-oxovalerate aldolase, *hsaG* acetaldehyde dehydrogenase, *hsaE* 2-hydroxypenta-2,4-dienoate hydratase, *hsd4B* 2-enoyl acyl-CoA hydratase, *choG* cholesterol oxidase

A scheme of the three *R. ruber kstD* ORFs and their genomic surroundings is depicted in Fig. 1 showing also the in silico predicted pP1, pP2, pP3, pP4 and pP5 promoters. The available programs (see “Methods”) yielded no putative promoter region just upstream either *kstD1* or *kstD2* ORFs, although it should be noted here that promoter prediction programs are not specific for Gram-positive species.

**Fig. 2:**
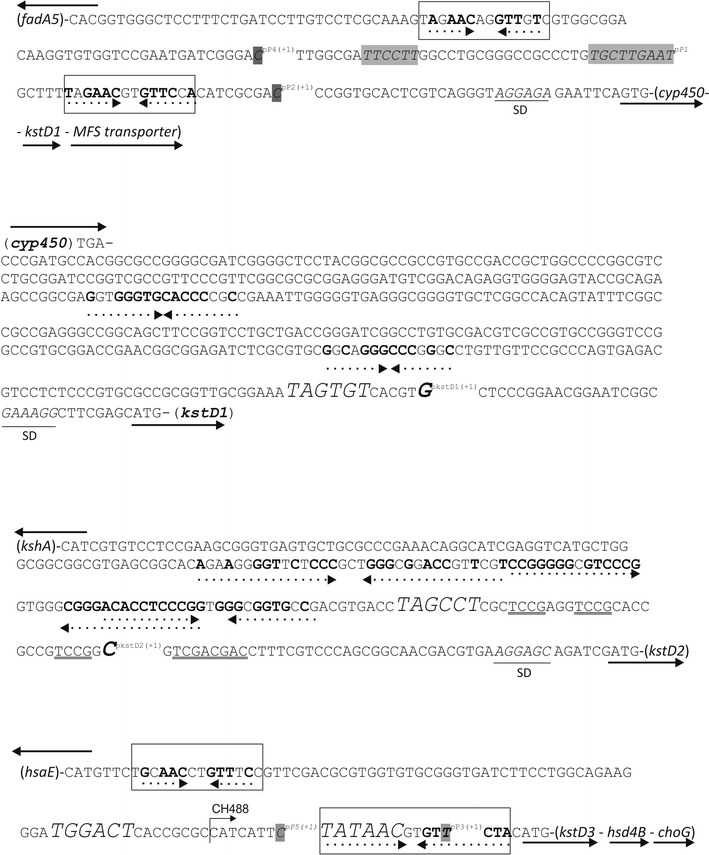
Sequence of regions upstream *kstD* ORFs. *Solid arrows* represent the orientation of the different ORFs from the initiation to the final codon. Sequences similar to *Mycobacterium* KstR binding sites (TnnAACnnGTTnnA) are within a *square*. Palindromic sequences appear in *bold* characters and *dotted underlined*. Shine Dalgarno (SD) sequences are in *italics* and underlined. The −10, −35 *boxes* are marked in *grey*. The in silico transcription initiation point of the putative pP1, pP2, pP3 and pP4 promoters are in italics and marked as pP(+1). The transcription start sites of *kstD1* and *kstD2* ORF obtained by the ARF-TSS method are shown in *bold italics* and marked as pKstD(+1). The TCCG repeats and the *Sal* box upstream *kstD2* are *double underlined*. Promoter signals similar to others described (e.g. *M. tuberculosis*) [55] appear in bigger size. Primer CH488 indicating the beginning of the minimum *kstD3* promoter is also shown

However, these programs detected putative promoters (pP1, pP2 and pP4) upstream the *cyp450* gene, lying in the intergenic region of the *fadA5*-*hyp* and *cyp450*-*kstD1*-*MFSt* opposite clusters. Flanking the pP1 putative promoter there are two palindromic sequences (TagAACagGTTgtc and TagAACgtGTTccA) (Fig. 2), one of them rather similar and the second one identical to the consensus binding region reported for the KstR regulatory protein of *Mycobacterium* (TnnAACnnGTTnnA) [48]. KstR and KstR2, two TetR family repressor regulators, have been found to control most of the steroid pathways in actinobacteria [48–50]. KstR is a highly conserved TetR family repressor that regulates the transcription of genes related to the upper and central pathway of cholesterol catabolism, namely the membrane transport of cholesterol, the degradation of the steroid side chain and the opening of the A and B rings [12, 48]. Upon binding to a 3-oxo-4-cholestenoic acid or to a CoA thioester cholesterol metabolite, KstR releases the DNA and allows transcription to begin [51, 52]. The KstR binding motif has been proved to be conserved within actinobacteria [48, 53].

The possible co-transcription of the *cyp450*-*kstD1*-*MSFt* ORF cluster to a polycistronic mRNA would come into contradiction with the transcription of a non-yet described short putative ORF (*pep*, Fig. 1) located in opposite sense in the 425 bp *cyp450*-*kstD1* intergenic region. This short ORF might code for a 35 amino acid peptide which shows a 98% amino acid identity with part of the hypothetical 78 aa protein RHRU231_750039 (*R. ruber*, 78 aa). Therefore, *kstD1* might be independently transcribed while pP1/pP2/pP4 putative promoters might be only involved in *cyp450* transcription. Moreover, a putative ribosome binding site (GAAAGG) was found 9 bp upstream the *kstD1* initiation codon (Fig. 2) that is identical to the proposed one for *sigA* of *R. ruber* TH [54].

In the case of the *kstD2* ORF, none of the online programs recognized a promoter consensus, although this region contains some quasi-palindromic sequences and a Shine-Dalgarno-like motif (AGGAGC) (Fig. 2).

There are two putative promoters for the *kstD3* ORF (pP3 and pP5, see Figs. 1 and 2) that lie in the intergenic region between *hsaE* and *kstD3* ORFs. The putative promoter pP3 contains the sequence TATAAC similar to the −10 consensus motif described for *M. smegmatis* promoters (T_100%_A_93%_T_50%_A_57%_A_43%_T_71%_) [55], and a −35 region (**T**G**GAC**T) that resembles the *E. coli* promoter consensus motif TTGACA. In this region, there are also a tandem of two putative KstR binding sequences around this promoter (TgcAACctGTTtcc and TatAACgtGTTctA), one quite similar and the other identical to the KstR binding consensus, in a similar way to the *cyp450* pP1/pP2 putative promoter (Fig. 2). The arrangement of these promoter and regulatory sequences, lying between opposite cluster genes, also occurs in the *R. ruber kstD1* region and it is similar to that found in *Mycobacterium* and *Rhodococcus jostii* RHA1 genomes [48, 56]. On the other hand, Shell et al. have recently described that the abundance of leaderless transcripts (that lack a 5′ UTR and a Shine-Dalgarno sequence and that begin with ATG or GTG) is a major feature of mycobacterial that accounts for around one-quarter of the transcripts [57]. *kstD3* could be a leaderless ORF: there is no evidence of a Shine-Dalgarno sequence in its 5′ region and the putative promoter is quite near to the ATG initiation codon. Moreover, as it will be stated later, the promoter of this intergenic region is functional in *Rhodococcus* but not in *E. coli,* a fact that has also been confirmed in the mycobacterial leaderless messenger translation [57].

### Promoter cloning and characterization

To go further in the characterization of the promoter regions of the *R. ruber kstD* ORFs, a promoter-test vector suitable for *R. ruber* strain Chol-4 was constructed. *R. ruber* is sensitive to apramycin so we chose the expression of a gene encoding this resistance as a proof of the promoter activity.

**Fig. 3:**
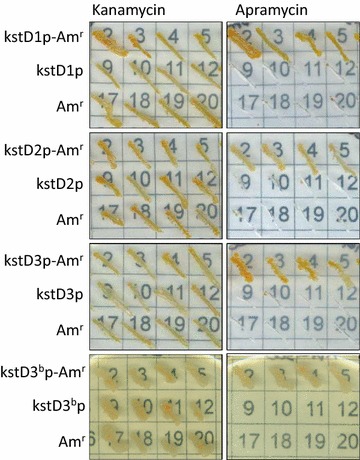
Comparative assessment of *R. ruber kstD* promoters. Cells of *R. ruber* strain Chol-4 harbouring different recombinant plasmids were grown in minimal medium supplemented with either 1.5 mM cholesterol (*kstD2, kstD3* or *kstD3* ^*b*^ promoters) or 2 mM AD (*kstD1* promoter) in the presence of either 200 µg/mL kanamycin or 300 µg/mL apramycin. kstDp: *R. ruber* cells harbouring pNVSP plasmids (*kstD* promoters cloned in pNVS vector). kstDp- Am^r^: *R. ruber* cells harbouring pNVSP-A plasmids (apramycin resistance gene fused to *kstD* promoters in pNVSP plasmids). Am^r^: pNVS vector containing the promoter less apramycin resistance gene

pNV119 [43], a *Nocardian* shuttle vector shown to replicate in *R. ruber* [24], was modified by adding the mcs of pSEVA351 that contains a transcriptional terminator in each extreme [41, 42]. The resulting pNVS plasmid (see Additional file 4) was used to study the activity of the Chol-4 putative promoter regions. The three *kstD* intergenic regions (Fig. 2) plus the first 21 bases of each *kstD* ORF were PCR amplified, transcriptionally fused to the apramycin resistance gene obtained from pIJ773 and cloned into the mcs of pNVS. The recombinant plasmids were introduced into *R. ruber* by electroporation and kanamycin resistant clones were selected. *R. ruber* clones, harbouring the plasmids pNVSP1-A, pNVSP2-A or pNVSP3-A, were then plated in minimal medium supplemented with either 1.5 mM cholesterol, 1.5 mM AD or 10 mM sodium acetate, and in the presence of either 200 µg/mL kanamycin or 300 µg/mL apramycin. The apramycin resistance gene (Am^r^) without any upstream promoter region was cloned in pNVS mcs generating the vector pNVSA that was used as a negative control. As a second control, a set of pNVSP vectors that contain every promoter region but do not carry the apramycin resistance gene was used. Figure 3 shows that only the cells harbouring the double system formed by a putative promoter and the apramycin resistance gene were able to grow on apramycin and kanamycin while cells harbouring the pNVSA or the pNVSPs vector were only able to grow in kanamycin plates. These results unambiguously confirm that all the three checked DNA regions contain *R. ruber* promoter sequences functionally active in the conditions used. On the other hand, *E. coli* harbouring the plasmids pNVSP1-A or pNVSP2-A were also able to grow in apramycin, in contrast with those harbouring pNVSP3-A (data not shown). Other actinobacteria promoters (e.g. some of *Mycobacterium* and *Streptomyces* spp.; [55, 58] are also not functional in *E. coli* strains; this fact could related to the occurrence of leaderless genes [57].

Although none of the online programs recognized a promoter consensus for the *kstD2* ORF, the promoter-less vector pNVS has enabled us to check the promoter activity of the intergenic regions. The construction of an improved version of this plasmid that allows a quantitative analysis of promoter strength is under work.

**Fig. 4:**
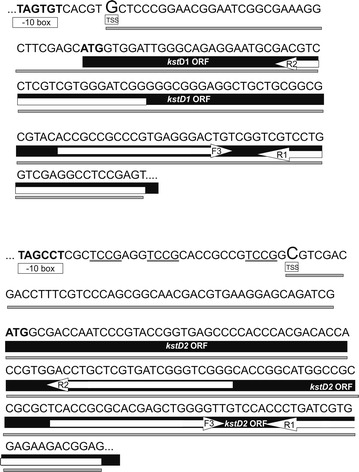
ARF-TSS analysis of *kstD* transcripts. The translation initiation codon ATG, the TSS identified by ARF-TSS and the proposed −10 box appear in *black*. R1, R2 and F3 primers are indicated. A *grey bar* shows the sequence obtained by ARF-TSS from the circularized cDNA and the subsequent PCR amplification with R2 and F3

To define the transcription start sites (TSS) of the *kstD* genes, the transcription start point protocol (ARF-TSS) indicated in “Methods” section was followed in *R. ruber* cells. We could conclude that the 5′ terminal base in *kstD1* messenger RNA is a G resulting from the transcription that starts 34 bp upstream the *kstD1* initiation codon. Similarly, the transcription start site of the *kstD2* gene is a C 48 bp upstream the *kstD2* initiation codon (Figs. 2, 4). However, this approach did not yield any result in the case of the *kstD3* gene. In order to better define the limits of the *kstD3* promoter, progressively shorter sections of the intergenic region, keeping the first 21 bases of *kstD3* ORF, were PCR amplified and transcriptionally fused to the apramycin resistance gene. As it can be seen in Figs. 2 and 3, just a minimum region of only 23 pb upstream the ATG of *kstD3* ORF (pNVSP3^b^-A vector) was enough to act as a promoter as the *Rhodococcus* cells harbouring this vector were able to grow on medium with either apramycin or kanamycin. This promoter region is partially similar to the putative pP3 and pP5 promoters mentioned before, containing the TATAAC sequence similar to the −10 consensus motif described for *Mycobacterium smegmatis* promoters (Fig. 1).

Putative −35 and −10 hexamers identification was based on the sequence of other actinobacteria promoters. The −10 region motif (TAGTGT) found 40 bases upstream the *kstD1* initiation codon is similar to the −10 consensus region described for *Mycobacterium tuberculosis* promoters (T_80%_A_90%_Y_60%_G_40%_A_60%_T_100%_) and identical to the T101 promoter described by Bashyam et al. in the same bacteria [55]. No sequence similar to any −35 motif was found in the *kstD1* region. The absence of −35 motifs seems to be a characteristic of actinobacterial genes [55, 58].

There is a putative −10 sequence (TAGCCT) in the intergenic region between *kshA* and *kstD2* that resembles the T6 promoter of *M. tuberculosis* (TAGGCT) [55]. However, it is a bit far away from the *kstD2* TSS. There is a repetitive motif TCCG in this region (Fig. 2) that also appears in the human *Sal* box element present in the 3′ terminal spacer of rDNA and that constitutes a termination signal for RNA polymerase I (**TCCGCAC**GG**GTCGAC**C**A**G) [59]. In this *kstD2* upstream region we found something similar: **TCCG**AGG**TCCGCAC**CGCCG**TCCG**GC**GTCGAC**G**A**C, where black-underlined letters marked the resemblance between them. Such promoter proximal terminators can appear also upstream of the transcription start site and in this case, they are described to positively affect transcription initiation and to prevent transcriptional interference by reading through of polymerases from the spacer that separates each rDNA repetition [60–62]. The biological significance of this motif in this intergenic *kstD2* region should be further determined.

### Transcriptional analysis of *kstD* genes in *R. ruber* strain Chol-4

The transcription of the three *kstD* genes found in *R. ruber* strain Chol-4 was analysed by RT-PCR of RNA samples prepared from cultures grown in either M457 mineral medium or LB medium supplemented with either AD or cholesterol as possible inductors.

**Fig. 5:**
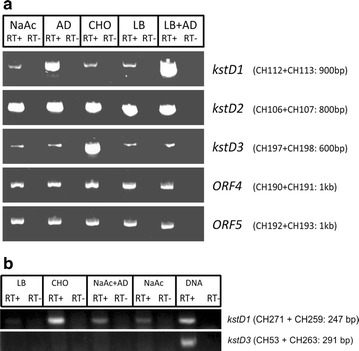
**a** Analysis of transcription products of *kstD* genes in *R. ruber* strain Chol-4 by specific RT-PCR amplification and agarose gel electrophoresis. RNA samples were isolated from cultures grown in: M457 minimal medium supplemented with sodium acetate (lane NaAc), AD (lane AD) or cholesterol (lane CHO) and LB medium supplemented with (lane LB + AD) or without AD (lane LB). RT—refers to negative controls (not incubated with retrotranscriptase) to exclude DNA contamination in RNA samples. Primers used in each PCR reaction and amplified fragment size are shown in *brackets*. As non-induced expression controls, the transcription of two *R. ruber* Chol-4 enzyme genes was followed in the same conditions: ORF4, that codes for a FAD-binding dehydrogenase D092_14375 (99% identity with the protein NCIB::WP_010594120.1 of *Rhodococcus* sp. P14) and ORF5 (D092_13875) that codes for a fumarate reductase (99% identity with NCIB::WP_010594021.1 of *Rhodococcus* sp. P14; see sequence in Additional file 6). the FAD-binding dehydrogenase D092_14375 and the fumarate reductase D092_13875. **b** RT-PCR assays of *R. ruber* Chol-4 *kstD1* and *kstD3* transcription carried out in *kstD2 R. ruber* mutant growing in: LB (lane LB) or M457 plus CHO (lane CHO), NaAc and AD (lane NaAc + AD) or NaAc (lane NaAc). Lane DNA shows the result of the control experiment: PCR made on *R. ruber* DNA

Control cultures were grown in either M457 supplemented with sodium acetate (2 g/L NaAc) or LB, both in the absence of any steroid. Using the specific primer pairs designed to search for the transcript of each different ORF (Additional file 2), we could show that all the *kstD* genes are transcribed in all the conditions used in our assays (Fig. 5) and that transcription of some of them is induced by cholesterol or AD. There are also other *Rhodococcus* metabolic genes reported to show a low-level constitutive transcription that can be strongly induced under the presence of a determinate substrate. For instance, phenol degradation genes in *R. erythropolis* are constitutively transcribed and also highly induced by phenol [63].

cDNA templates concentrations were adjusted to the same value in RT-PCR experiments, so we can consider the differences observed in the thickness of some amplification bands to be meaningful (Fig. 5). Specific amplification of *kstD1* cDNA was higher in AD induced than in non-induced cultures, while amplification of *kstD3* cDNA was higher in cultures induced with cholesterol as compared to the other conditions assayed. So we can conclude that these two genes, although constitutively transcribed, they can also be additionally induced by the presence of AD (*kstD1*) and cholesterol (*kstD3*). The induction of *kstD* genes by cholesterol or AD has also been reported in other microorganisms: a 3.3 expression ratio (cholesterol/pyruvate) was reported for one *kstD* in *R. jostii* RHA1 (*ro04532*) [64, 65], while other putative *kstD* genes of the same strain (e.g. *ro02483*, *ro05798* and *ro05813*) were up-regulated in 7-ketocholesterol but not in cholesterol [64]; the main *kstD* in *M. smegmatis* (*MSMEG_5941*) was 13-fold up-regulated in cholesterol respect to glycerol [12]; a 1.8, 4.1 or 1.2 -fold up-regulation (AD/glycerol) was found for *kstD1*, *kstD3* and *kstD2* genes respectively in *Mycobacterium neoaurum* [66]. These differences in regulation among the different *kstD* genes within the same strain highlight the view that KstD proteins may be acting in different metabolic steps and/or pathways, each one having a particular catalytic role.

The amplification of *R. ruber* strain Chol-4 *kstD2* cDNA yielded thick amplification bands in all cases (Fig. 5), which clearly leads to propose a constitutive expression of *kstD2*. In a similar way, two putative *kstD* genes in *R. jostii* RHA1 genome (*ro90203* and *ro09040*, belonging to the KstD2-branch of the KstD phylogenetic tree) [20] were expressed but not up-regulated neither in cholesterol nor in 7-ketocholesterol, when compared to pyruvate [64].

The expression profile of *R. ruber kstD1*, *kstD2* and *kstD3* genes was determined by real-time PCR. Taking as 1 the expression levels on sodium acetate (unexposed steroid culture), the values obtained for the expression of the three genes were: 7.6, 2.0 and 240.5-fold for *kstD1*, *kstD2* and *kstD3*, respectively, in cultures grown in cholesterol; and 13.6, 0.7 and 0.6-fold for *kstD1*, *kstD2* and *kstD3*, respectively, in cultures grown in AD.

The particular organization of *R. ruber* Chol-4 *kstD1* and *kstD3* genes (Fig. 1) opens the possibility that polycistronic *kstD* mRNAs could be synthesized by the co-transcription of the *cyp450*-*kstD1*-*MFS transporter* and *kstD3*-*hsd4B*-*choG* gene clusters. The results of the RT-PCR experiments did not show the occurrence in AD culture medium of either *cyp450*-*kstD1* or *kstD1*-*MFS transporter* RNA sequences, indicating that *kstD1* gene is independently transcribed.

In contrast, co-transcripts from both *kstD3*-*hsd4B* ORFs and *hsd4B*-*choG* ORFs could be amplified from cultures grown in the presence of cholesterol, strongly suggesting that *kstD3*-*hsd4B*-*choG* ORFs are co-transcribed into a polycistronic mRNA (Fig. 1). This group of three genes are also described to be co-transcribed in *R. erythropolis* [27].

The adjacent location of *kstD3*-*hsd4B* ORFs is highly conserved among rhodococci. The *hsd4B* ORF encodes a 2-enoyl acyl-CoA hydratase involved in the β-oxidative cycle of the C-17 cholesterol side chain [65]. The *choG* ORF encodes an extracellular cholesterol oxidase that it is involved in the first step of cholesterol catabolism that implies its conversion to 4-cholesten-3-one [27, 47, 67–69]. All this suggests that *kstD3, hsd4B* and *choG* genes are mainly involved in the steroid catabolism.

In this work we showed that *kstD2* is constitutively transcribed, while *kstD1* and *kstD3* are also constitutively but faintly transcribed, although they can be highly induced by AD or CHO. In a previous work [24], we reported the construction of a *kstD2* deletion mutant of *R. ruber* that is unable to grow in minimal medium supplemented with AD. The question is why KstD1 and/or KstD3 cannot substitute KstD2 allowing the *kstD2* deletion mutant to grow on AD. To partially advance in this subject, RT-PCR experiments were also performed on RNA from the *kstD2 R. ruber* mutant to check the expression of the other *kstD* genes (Fig. 5b).

The results showed that the transcription pattern of the *kstD* genes of the mutant is far different than that of the same genes in the wild type. Namely, *kstD1* gene in the mutant was constitutively and slightly transcribed and its expression is induced in CHO. A more noticeable change affects to the *kstD3* gene, which is not transcribed at all in any of the conditions used. These data reveal a complex relationship among the KstD enzymes and their expression control mechanisms. Modification of the *kstD* transcription levels of genes remaining in the cell have also been described in a *M. neoaurum kstD* mutant: the transcription ratio of the *kstD1* ORF (similar to the *kstD3* ORF from *R. ruber*) in AD induced cultures respect to glycerol cultures increases from 1.8 (in the wild type strain) to 2.7-fold (in the *kstD3* *M. neoaurum* mutant strain) [66].

An appealing conclusion of that complex situation is that the three KstD proteins of *R. ruber* strain Chol-4 may be differentially involved in distinct pathways of steroid degradation, and that their expression could also be differentially and specifically controlled.

### Heterologous expression of KstD1, KstD2 and KstD3 of *R. ruber* strain Chol-4

The three *R. ruber kstD* genes (*kstD1*, *kstD2* and *kstD3*) were cloned into the pTip-QC1 expression vector. *R. erythropolis* CECT3014 cells were electroporated with these constructions and clones harbouring each of those recombinant plasmids were isolated. Expression of the KstD proteins from these vectors in the CECT3014 transformed cells was followed by SDS-PAGE analysis. Molecular weights were 54.8, 60.8 and 61.8 kDa for KstD1, KstD2 and KstD3 respectively (Additional file 5). Cell-free extracts of cultures grown from these clones were used for the analysis of KstDs activities, and the kinetic parameters of the heterologously expressed KstDs from *R. ruber* were followed for different substrates (Table 1; see also Additional file 3 for substrates structure). Control cell extract from the *R. erythropolis* culture harbouring an empty pTip-QC1 vector yielded none or very low basal levels when acting on all the substrates used in the assay and were taken into account for final activities.

**Table 1:** Substrate profiles of *R. ruber* KstDs expressed an analyzed in cell-free extracts of *R. erythropolis*

Substrate	KstD1				KstD2				KstD3			
	Rel. act %	Km (µM)	RCE	RCE/RCE_AD_	Rel. act %	Km (µM)	RCE	RCE/RCE_AD_	Rel. act %	Km (µM)	RCE	RCE/RCE_AD_
AD	100.0 ± 11.6	34.2 ± 3.8	2.92	1.00	100.0 ± 11.8	40.1 ± 8.7	2.60	1.00	nd	nd		
9OHAD	107.4 ± 13.4	22.1 ± 7.0	4.84	1.66	29.8 ± 6.5	543.2 ± 77.8	0.05	0.02	nd	nd		
4BNC	52.3 ± 7.4	76.1 ± 15.7	0.69	0.24	30.8 ± 7.2	38.2 ± 6.6	0.81	0.31	nd	nd		
Prog	89.7 ± 7.9	27.9 ± 9.5	3.22	1.10	182.0 ± 7.0	33.8 ± 4.9	5.38	2.07	18.6 ± 3.7	43.6 ± 2.9	0.42	0.78
Cort	20.3 ± 5.8	161.6 ± 7.6	0.13	0.04	19.0 ± 3.0	374.3 ± 74.5	0.05	0.02	nd	nd		
Tes	134.6 ± 22.6	28.8 ± 7.4	4.67	1.60	233.3 ± 23.3	107.9 ± 18.5	2.16	0.83	nd	nd		
19OHAD	39.0 ± 10.6	368.8 ± 102.4	0.11	0.04	24.6 ± 7.8	347.4 ± 41.7	0.07	0.03	nd	nd		
DOC	67.0 ± 7.4	21.6 ± 4.5	3.10	1.06	124.2 ± 17.2	42.5 ± 5.8	2.92	1.12	21.6 ± 8.8	111.3 ± 4.0	0.19	0.36
5α-Tes	nd	nd			75.2 ± 4.8	24.57 ± 7.8	3.06	1.18	100.0 ± 21.9	181.1 ± 42.6	0.55	1.00

Rel. act: relative activity values. Enzyme activities are expressed as percentage of activity of AD (for KstD1 and KstD2 with 3.2 U/mg and 1.4 U/mg respectively) or 5α-tes (for KstD3 with 0.3 U/mg) that were set as 100%

*nd* enzyme activity was not detected for this substrate, *RCE* relative catalytic efficiency given by the ratio Rel. act/km, *Prog* progesterone, *Cort* corticosterone, *Tes* testosterone, *5α-tes* 5-alpha-testosterone, *19OHAD* 19-hydroxy-4-androstene-3,17-dione, *DOC* deoxycorticosterone, *4-BNC* 4-pregnen-3-one-20β-carboxylic acid

The substrate profile of KstD1 showed a clear preference to 9OHAD and testosterone, followed by progesterone, Deoxy corticosterone (DOC) and AD (Table 1). All these compounds display a keto group at C3, a C4-C5 double bond and an electronegative side-chain at C17 (Additional file 3). When comparing to the substrate preference order of *R. erythropolis* SQ1 KstD1 (Prog > 9OHAD, AD > 5α-Tes > BNC > 11βCort), some details highlights: (i) KstD1_SQ1_ has a relative catalytic efficiency (RCE) on progesterone 3.4 times higher than on AD, a very big difference to the ratio 9OHAD/AD (1.6) showed by *R. ruber* KstD1 (Table 1); (ii) *R. ruber* KstD1 is not active on 5α-Tes in contrast to KstD1_SQ1_.

The order of substrate preference of *R. ruber* KstD2 placed progesterone in the first position, followed by 5α-Tes, DOC, AD and testosterone (Table 1), displaying a substrate profile similar to KstD2_SQ_1 enzyme (Prog > AD > 5α-Tes > 9OHAD, BNC > 11βCort). It is noteworthy the similarity of both KstD profiles, having in mind that they have been expressed in different cellular context (a *Rhodococcus* strain, and *E. coli*). *R. ruber* KstD2 has a broader range of substrates than *R. ruber* KstD1 as it can act on all the KstD1 substrates and also on 5α-Tes that contains a saturated A ring (Table 1).

*Rhodococcus ruber* KstD3 did not show any activity at all when acting on AD or 9OHAD, which are considered the natural substrates for KstD enzymes, but it showed the highest activity when using 5α-Tes as substrate followed by progesterone and lastly DOC (Table 1). *R. ruber* KstD3 has a very narrow substrate range similarly to the *R. erythropolis* SQ1 and *M. tuberculosis* H37Rv isoforms, being the A-ring saturated 5α-Tes the preferred substrate for all KstD3 enzymes (Table 1, [20]). However, KstD3 affinity for 5α-Tes differs from 33–36 µM in the cases of KstD3_H37RV_ and KstD3_SQ1_ respectively, to 181 µM in *R. ruber* KstD3 (Table 1). Despite this low affinity for 5α-Tes, this is the best substrate for the *R. ruber* enzyme among those that were assayed (Table 1).

On the other hand, although it has been proposed that only steroids carrying a small or no aliphatic side chain at C-17 are suitable substrates for KstD3 [20], a minor activity, not very different to that obtained with progesterone, has been observed in the assays of *R. ruber* KstD3 enzyme on DOC (Additional file 3; Table 1). *R. ruber* KstD3 seems to be more related to the cholesterol metabolism than to the AD metabolism [24] and then it could be acting on some not yet neatly defined intermediaries of the bacterial cholesterol metabolism.

None of the three *R. ruber* KstD proteins displayed detectable activity on 4-cholestene-3-one, 5α-cholestane-3-one or 5β-androstane-3,17-dione (5β-AD), ADD, cholesterol, cholestenone, cholic acid, DHEA, ergosterol, stigmasterol, β-estradiol, sodium deoxycholate or 5-pregen-3-β-nolone. Therefore, these KstDs catalyse preferentially 4-ene-3-oxosteroids.

### Molecular modelling of KstD1 of *R. ruber* strain Chol-4

The sequence of the three KstDs of *R. ruber* were described previously [24]. In an attempt to go deeper inside the reasons of the catalytic and kinetic differences among some of these enzymes, we performed protein sequence analysis and modelling studies on the *R. ruber* KstDs using different approaches. The published three-dimensional structure and the catalytic mechanism proposed for KstD1 from *R. erythropolis* SQ1 has been used as a suitable model [21, 70]. The I-Tasser and PredictProtein programmes predicted that none of the three *R. ruber* KstDs contain sulphur bridges or transmembrane segments.

**Fig. 6:**
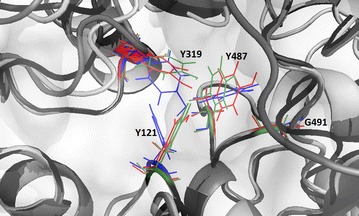
Modelling of the active site of KstDs. The orientation of the four key residues in the KstD binding pocket is shown. They are superposed and depicted in different colours: *green* for KstD1, *blue* for KstD2 and *red* for KstD3. Only KsD1 residues are named, their counterpart homologues residues of KstD2 and KstD3 being listed in Tables 2 and 3

The catalytic mechanism of *R. erythropolis* SQ1 KstD1 is based in the keto-enol tautomerization of the substrate caused by Tyr487 and Gly491 residues that increases the acidity of the C2 hydrogen atoms of the substrate. Then Tyr119 and Tyr318 capture the axial β-hydrogen from C2 as a proton whereas the FAD molecule accepts the axial α-hydrogen from the C1 atom of the substrate as a hydride ion [21, 70]. Tables 2 and 3 collects the residues involved in the active site of KstD1_SQ1_ described for Rohman et al. [21] and the homologue residues of the three KstDs from *R. ruber* found by COBALT programme. The four key residues: Tyr119, Tyr318, Tyr487 and Gly491 of the KstD1_SQ1_ active site have a counterpart residue in the *R. ruber* KstDs. However, the I-TASSER model prediction of these *R. ruber* enzymes shows that the orientations of the side chain of the tyrosine residues differ within the catalytic pocket site being specific of each enzyme. The variation in orientation of the key residues inside the catalytic poked is shown in Fig. 6. Particularly, the orientation of KstD3 Y354 and Y530 residues is almost opposite to that of its homologous KstD1 and KstD2 residues. Moreover, amino acid Y128 from KstD2 has a position highly separated compared to its homologous from KstD1 (Y121) and KstD3 (Y119). These differences could justify the different affinity and catalytic properties among the three KstDs. The current shortage of crystalline structures of these proteins greatly limit more detailed conclusions.

**Table 2:** Homologous residues of KstDs implicated in the catalytic pocket attending to Rohman et al. [21]

KstD1-SQ1	KstD1-ruber	KstD2-ruber	KstD3-ruber
S52	G53	A59	G50
F116	W118	Y125	Y116
*Y119*	*Y121*	*Y128*	*Y119*
F294	F295	P337	F327
V296	L297	V339	S330
*Y318*	*Y319*	*Y366*	*Y354*
I352	T356		I395
I354	V358	F405	P397
L447	L447	L499	L490
*Y487*	*Y487*	*Y539*	*Y530*
P490	G490	A542	P533
*G491*	*G491*	*G543*	*G534*
V492	N492	A544	A535
P493	P493	T545	T537

Italic files contain the four key residues of the KstD1 active site reported for *R. erythropolis* SQ1: Tyr119, Tyr318, Tyr487 and Gly491

**Table 3:** PredictProtein prediction of the secondary structure and solvent accessibility in % of the three KstDs from *R. ruber*

Structure	KstD1	KstD2	KstD3
Strand	12.72	11.88	64.74
Loop	63.99	64.01	14.38
Helix	23.29	24.11	20.88

Accessibility	KstD1	KstD2	KstD3
Exposed	24.1	21.81	22.11
Buried	67.7	71.63	70.5
Intermediate	8.22	6.56	7.54

A redundancy of KstD and Ksh enzymes have been described in the actinobacteria genomes [20, 24, 64, 70]. This redundancy could provide the cell with a bigger metabolic versatility and a fine-tuned response to the challenging environment. In the case of KstD enzymes, three homologues have been found in *R. erythropolis* strain SQ1 and in *M. neoaurum* that displayed different substrate preferences and that could be involved in different metabolic steps in a strain-dependent way [20, 32, 66].

Even more, it has been shown recently that mutations in the KstDs provokes a different ADD/AD molar ratio [71] and that environmental factors such as an increase of temperature can inhibit the KstD/Ksh action [72] on phytosterol in *Mycobacterium* sp. These multiplicity and versatility give to these enzymes a substantial role in the catalytic dehydrogenation of several related steroid molecules and pose many difficulties to clarify the particular role and way of acting of every single KstD.

Our results suggest that both KstD1 and KstD2 of *R. ruber* could act in the conversion of AD to ADD being KstD1 mainly involved in the 9OHAD to 9OHADD conversion, in a similar way to what has been described in *R. erythropolis* SQ1 [32]. However, there are differences between these two strains, as the necessity of a double *kstD1* and *kstD2* mutation to prevent the growth in AD in the case of *R. erythropolis* SQ1 [32] or *R. rhodochrous* DSM43269 [73], while the same effect is obtained by the single *kstD2* deletion in *R. ruber* strain Chol-4 [24], suggesting that this last mutation affects in some way the activity of the KstD1 protein.

*kstD3* ORF occurs in the *R. ruber* genome in a quite conserved location within *Rhodococcus* species, clustered with *hsd4B* (which encodes a 2-enoyl acyl-CoA hydratase proposed to be involved in cholesterol side-chain shortening) [65] and *choG* (coding for a cholesterol oxidase that converts cholesterol into cholestenone) [27, 47, 68]. Recently it has been proposed that sterols can be catabolised in *R. equi* USA-18 by two partially different pathways, namely via AD or via Δ1,4-BNC, that converge in the intermediary 9OHADD [74]. Given its substrate preference, the fact that the growth in AD is independent of the KstD3 activity while the growth in cholesterol needs the presence of either KstD2 or KstD3 in *R. ruber* [24], lead us to suggest that KstD3 may be involved in an alternative AD-independent cholesterol catabolic pathway.

## Conclusions

To sum up, this study provides biochemical and genetic insights into the three KstD proteins found in *R. ruber*. The kinetic differences between the three KstDs suggest that each enzyme could act on different steps of the steroid catabolic routes. Both KstD1 and KstD2 could be involved in the AD catabolism while KstD1 would have a preference for 9OHAD and KstD2 for progesterone. KstD2 seems to be a more versatile enzyme than KstD1 in *R. ruber* as it can act also on saturated steroid substrates such as 5α-Tes. On the other hand, the narrower range of substrates for KstD3 and its preference for saturated steroid made this enzyme different to KstD1 and KstD2 and suggest that it may be involved in AD-independent steroid catabolism. The differences found in the orientation of catalytic residues of each KstD within the binding pocket site could explain the substrates preferences of each enzyme.

The promoter regions that support transcription of *kstD* genes have been cloned and functionally identified. The three promoter boxes contain different expression patterns, from the TCCG motif found in the *kshA*-*kstD2* ORF intergenic region to the KstR boxes in the *hsaE*-*kstD3* intergenic region. Moreover, *kstD3* ORF was transcribed as a polycistronic *kstD3*-*hsd4B*-*choG* mRNA and was induced in cholesterol growing media, reinforcing the role of KstD3 in the cholesterol metabolism. Potential functions of *R. ruber* strain Chol-4 KstDs in other steroid pathways remain to be elucidated.

## Additional files

**Additional file 1.** Bacterial strains and plasmids used in this work.

**Additional file 2.** Primers used in this work. Restriction sites are marked in bold.

**Additional file 3.** Structure of the steroids used in this work.

**Additional file 4.** Scheme of the pNVSP vectors construction. The shuttle vector pNV119 was modified to include a mcs from pSEVA351 (pNVS) and after that coupled with the apramycin resistance ORF. Intergenic regions containing the putative *kstD* promoters were cloned in the mcs to obtain pNVSP vectors.

**Additional file 5.** Expression of KstDs from ptip-QC1 vectors in induced *R. erythropolis* CECT3014 cells. SDS-PAGE analysis on a 12.5% gel was performed using 10 µg of the cell-free extracts. The band corresponding to KstD overexpression is marked with a rectangle. Precision plus protein standard from Bio-Rad was used as size marker.

**Additional file 6.** *R. ruber* strain Chol-4 ORF4 and ORF5 protein sequences.

## Abbreviations

AD: 4-androstene-3,17-dione
ADD: 1,4-androstadiene-3,17-dione
5β-AD: 5β-androstane-3,17-dione
9OHAD: 9α-hydroxy-4-androstene-3,17-dione
9OHADD: 9α-hydroxy-1,4-androstadiene-3,17-dione
4-BNC: 4-pregnen-3-one-20β-carboxylic acid
5α-Tes: 17β-hydroxy-5α-androstan-3-one
DOC: deoxy corticosterone
ORF: open reading frame
DCPIP: 2,6-dichlorophenol-indophenol
KstD: 3-ketosteroid-Δ^1^-dehydrogenase
LB: Luria-Bertani
mcs: multiple cloning site
TSS: transcriptional start site
