# Supplementary material for: Functional differentiation of 3-ketosteroid Δ1-dehydrogenase isozymes in Rhodococcus ruber strain Chol-4
Source: Microb Cell Fact. 2017 Mar 14;16:42. doi: 10.1186/s12934-017-0657-1 (PMC5348764; doi:10.1186/s12934-017-0657-1)
Supplement: Supplementary file 1 — Additional file 1. Bacterial strains and plasmids used in this work. [file 12934_2017_657_MOESM1_ESM.docx]

**Additional file 1** Bacterial strains and plasmids used in this work

| **Bacteria and plasmids** | Description | **Reference** |
| --- | --- | --- |
| *Rhodococcus ruber* strain Chol-4 | Wild type phenotype, Nal^r^ | CECT7469 [25] |
| *Rodococcus erythropolis* strain *3014* | Wild type phenotype, Nal^r^ | CECT3014 |
| *E. coli* DH5α | F’ *endA1 hsdR17* (r_K_^-^m_K_^+^) *glnV44 thi-1 recA1 gyrA*(Nal^r^) *relA1* Δ(*lacIZYA-argF*) *U169deoR* (φ80*dlac*Δ(*lacZ*)*M15*) | Laboratory collection |
| pBluescript II KS + | Cloning Vector, Ap^r^ | Stratagene |
| pGEM-T Easy Vector | Cloning vector, Ap^r^ | Promega |
| pTip-QC1 | Expression *E. coli-Rhodococcus* shuttle vector, Ap^r^, P_TIPA_ Chl^r^REPAB (pRE2895) | [30] |
| pTip-*kstD1* | Expression plasmid harbouring *kstD1* (*Nde*I-*Bgl*II, 1.5kb) | This work |
| pTip-*kstD2* | Expression plasmid harbouring *kstD2* (*Nde*I-*Bgl*II, 1.6kb) | This work |
| pTip-*kstD3* | Expression plasmid harbouring *kstD3* (*Nde*I-*Bam*HI, 1.7kb) | This work |
| pNV119 | *Nocardia-E. coli* replicative shuttle vector, Km^r^ | [43] |
| pSEVA351 | Cloning vector, Cm^r^ | [41,42] |
| pNVS | *Nocardia-E. coli* replicative shuttle vector containing Teminator 1-mcs-terminator 0 from pSEVA351, Km^r^  (*Nhe*I-*Pci*I, 0.4Kb) | This work |
| pIJ773 | Cloning vector carrying the Am^r^ gene | [44] |
| pNVSP1 | pNVS containing *kstD1* promoter region and the sequence of the first seven amino acids in the mcs.  (*Xba*I-*Pst*I, 0.4Kb) | This work |
| pNVSP2 | pNVS containing *kstD2*promoter region and the sequence of the first seven amino acids in the mcs.  (*Xba*I-*Pst*I, 0.2Kb) | This work |
| pNVSP3 | pNVS containing *kstD3* promoter region and the sequence of the first seven amino acids in the mcs.  (*Xba*I-*Pst*I, 0.1Kb) | This work |
| pNVSP3^b^ | pNVS containing *kstD3* minimal promoter region and the sequence of the first seven amino acids in the mcs.  (*KpnI*-*Pst*I, 0.05Kb) | This work |
| pNVSP1-A | pNVSP1 in frame to *Am^r^* gene  (*Nru*I-*Hind*III, 0.8Kb) | This work |
| pNVSP2-A | pNVSP2 in frame to *Am^r^* gene  (*Nru*I-*Hind*III, 0.8Kb) | This work |
| pNVSP3-A | pNVSP3 in frame to *Am^r^* gene  (*Nru*I-*Hind*III, 0.8Kb) | This work |
| pNVSP3^b^-A | pNVSP3^b^ in frame to *Am^r^* gene  (*Nru*I-*Hind*III, 0.8Kb) |  |
| pNVSA | A derivative of pNVSP1-A with the promoter P1 region deleted | This work |
