# Supplementary material for: Functional differentiation of 3-ketosteroid Δ1-dehydrogenase isozymes in Rhodococcus ruber strain Chol-4
Source: Microb Cell Fact. 2017 Mar 14;16:42. doi: 10.1186/s12934-017-0657-1 (PMC5348764; doi:10.1186/s12934-017-0657-1)
Supplement: Supplementary file 2 — Additional file 2. Primers used in this work. Restriction sites are marked in bold. [file 12934_2017_657_MOESM2_ESM.docx]

**Additional file 2**. Primers used in this work. Restriction sites are marked in bold.

| **Primer** | **Sequence** | **PCR conditions** | **Use** |
| --- | --- | --- | --- |
| 3exF | AA**CATATG**GTGGATTGGGCAGAGGAAT | Tm 60 °C, 2 min, 30 cycles | *kstD1 Nde*I-*Bgl*II amplification for cloning and expression, 1.5 kb |
| 3exR | **AGATCT**GGGCGGCCGGCGTCGTCCTTAC |  |  |
| 2exF | AA**CATATG**GCGACCAATCCCGTACCGG | Tm 65 °C, 2 min, 30 cycles | *kstD2 Nde*I-*Bgl*II amplification for cloning and expression, 1.6 kb |
| 2exR | AA**AGATCT**TCAGCGGGACTTCGCGGCGTCC |  |  |
| 1exF | AA**CATATG**ATCAAGCAGGAATACGACA | Tm 55 °C, 2 min, 30 cycles | *kstD3 Nde*I-*Bam*HI amplification for cloning and expression, 1.7 kb |
| 1exR | AA**GGATCC**TCAGTGCTGTTCGACGGTCTCG |  |  |
| CH112 | CATCGACGACGCCCTGACCTACTA | Tm 55 °C, 1 min, 25 cycles | *kstD1* amplification in RT-PCR, 900 bp |
| CH113 | GAGCGTGTCGGCGGTCTTCC |  |  |
| CH106 | CCAAGGGCTATTCCGACTACCA | Tm 55 °C, 1 min, 25 cycles | *kstD2* amplification in RT-PCR, 800 bp |
| CH107 | GAAAAGCCCGCCGCCGAAGATGTA |  |  |
| CH197 | GTGCGCATCTGCTCGTTGTGTT | Tm 55 °C, 1 min, 25 cycles | *kstD3* amplification in RT-PCR, 600 bp |
| CH198 | GCCGGCAAGATCGACACCTACAT |  |  |
| CH190 | CGACGGCAGCTGTACGAGACCT | Tm 60 ºC, 1 min, 25 cycles | ORF 4 amplification in RT-PCR, 1000 bases |
| CH191 | AACTGCCCGGGACGACCTTG |  |  |
| CH192 | GCTCGACACCACACCGCTGAA | Tm 60 ºC, 1 min, 25 cycles | ORF 5 amplification in RT-PCR, 1000 bases |
| CH193 | GCTCTCGCCGCGGTGGTATT |  |  |
| CH344 | ACCGGAAGCTGCCGGCCCT | Tm 58 °C, 0.5 min, 25 cycles | *Cyp450-kstD1* coexpression (RT-PCR) 242bp |
| CH345 | TGCCCCGTCGAGCACTGACC |  |  |
| CH505 | GACATCGAGTTCACGGCCTA | Tm 60 °C, 1min, 30 cycles | *kstD1* amplification in RT-qPCR, 50 bp |
| CH506 | GGAGCCTTGCCGAAGTAGTC |  |  |
| CH507 | TCCTTCATCGTCGACCACAC | Tm 60 °C, 1min, 30 cycles | *kstD2* amplification in RT-qPCR, 69 bp |
| CH508 | CTGGCCGAACGACATGTAGT |  |  |
| CH509 | GCTACGACCACTACTACGGC | Tm 60 °C, 1min, 30 cycles | *kstD3* amplification in RT-qPCR, 92 bp |
| CH510 | CACCACCTTGATCGCGTAGA |  |  |
| CH518 | GGCATCTCACTGGGCGAAT | Tm 60 °C, 1min, 30 cycles | *D092_14375* reference gen in RT-qPCR, 69 pb |
| CH519 | CCAGGGAGACGGGACAGTA |  |  |
| CH371 | GCACATGGCCGGCGCGTAA | Tm 58 °C, 0.5 min, 25 cycles | *kstD1 – MFS transporter*  coexpression (RT-PCR) 210 bp |
| CH372 | GGTCGAGGGTGCCCGTTCGG |  |  |
| CH88 | CTCCGTCCGCCGAACAGAAAG | Tm 58 °C, 0.5 min, 25 cycles | *kstD3 –hsd4B* coexpression (RT-PCR) 210 bp |
| CH337 | CGGTGGCGAAGGTGGGCAG |  |  |
| CH338 | TCCTCACCCTCGCCTGACCC | Tm 55 °C, 0.5 min, 25 cycles | *hsd4B*-choG coexpression (RT-PCR) 158bp |
| CH339 | GGGGTTCGGTGCGAATCGTC |  |  |
| CH259 | **AACATA**TGGTGGATTGGGCAGAGGAAT | Tm 50 °C, 0.5 min, 25 cycles | *kstD1* amplification in RT-PCR, 247bp |
| CH271 | **GATAT**CGACGACGGCATGGTAGTAGGT |  |  |
| CH53 | GGTCGCCGTAGTAGTGGTCGTA | Tm 50 °C, 0.5 min, 25 cycles | *kstD3* amplification in RT-PCR, 291bp |
| CH263 | **GGATCC**GCGACGGGCCCGGCGAGAACAT |  |  |
| CH348 | **TCGCGAGCTAGC**AGGGCGCGCCCAGCTGTC | Tm 55 °C, 0.5 min, 30 cycles | *T0-MCS-T1* of pSEVA351  amplification for cloning, 415 bp |
| CH349 | **AGTACTACATGT**AAATCGTAATTATTGGGG |  |  |
| CH353 | **GGTACCGCATGCTCTAGA**CTGACCCGATGCCACGGCGCC | Tm 58 °C, 0.5 min, 30 cycles | Promoter region of *kstD1* and its first 21 nucleotides of coding sequence, amplification for cloning, 478 bp |
| CH354 | **TTTAAACTGCAGTCGCGA**TTCCTCTGCCCAATCCACCA |  |  |
| CH355 | **GGTACCGCATGCTCTAGA**CATCGTGTCCTCCGAAGCGG | Tm 58 °C, 0.5 min, 30 cycles | Promoter region of *kstD2* and its first 21 nucleotides of coding sequence, amplification for cloning,285 bp |
| CH356 | **TTTAAACTGCAGTCGCGA**CGGTACGGGATTGGTCGCCA |  |  |
| CH357 | **GGTACCGCATGCTCTAGA**CCATGTTCTGCAACCTGTTT | Tm 58 °C, 0.5 min, 30 cycles | Promoter region of *kstD3* and its first 21 nucleotides of coding sequence, amplification for cloning, 142 bp |
| CH358 | **TTTAAACTGCAGTCGCGA**GTATTCCTGCTTGATCATGT |  |  |
| CH488 | **GGTACCGCATG**CATCATTCTATAACGTGT | Tm 58 °C, 0.5 min, 30 cycles | In combination with CH402: last 23 pb of the promoter region of *kstD3* and its first 21 nucleotides of coding sequence, amplification for cloning, 856 bp |
| CH423 (R1) | **[Phos]** ACTCGGAGGCCTCGACCAGGAC | RT 55 °C | 5’-phosforilated primer for reverse transcription TSS *kstD1* |
| CH424 (R2) | CCGATCCCACGACGAGGACGTC | Tm 55 °C, 0.5 min, 30 cycles | Primers for PCR amplification of circularized *kstD1* cDNA, TSS |
| CH425 (F3) | ACCGCCGCCCGTGAGGGACTGT |  |  |
| CH427 (R1) | **[Phos]** CTCCGTCTTCTCCACGATCAGGG | RT 55 °C | 5’-phosforilated primer for reverse transcription TSS *kstD2* |
| CH428 (R2) | CCCGACCCGATCACGAGCAGGT | Tm 55 °C, 0.5 min, 30 cycles | Primers for PCR amplification of circularized *kstD2* cDNA, TSS |
| CH429 (F3) | TCACCGCGCACGAGCTGGGGTT |  |  |
| CH443 (R1) | **[Phos]** CGGAATCCACACGCCGCCGCCCGA | RT 58 °C | 5’-phosforilated primer for reverse transcription TSS *kstD3* |
| CH431 (R2) | GGTCAGCGCGGCGGTCATTC | Tm 56 °C, 0.5 min, 30 cycles | Primers for PCR amplification of circularized *kstD3* cDNA, TSS |
| CH432 (F3) | GCGTCGTCCTGGTCGAGAAGGC |  |  |
| CH401 | **CCGCGGTCTAGATCGCGA**GTGCAATACGAATGGCGAAAA | Tm 58 °C, 0.5 min, 30 cycles | *Am^r^*  (*Nru*I-*Hind*III) amplification for cloning 804pb |
| CH402 | **GGTACCAGATCTAAGCTT**TCATCAGCCAATCGACTGGCGAGC |  |  |
