# Supplementary figures and images for: Functional differentiation of 3-ketosteroid Δ1-dehydrogenase isozymes in Rhodococcus ruber strain Chol-4
Source: Microb Cell Fact. 2017 Mar 14;16:42. doi: 10.1186/s12934-017-0657-1 (PMC5348764; doi:10.1186/s12934-017-0657-1)

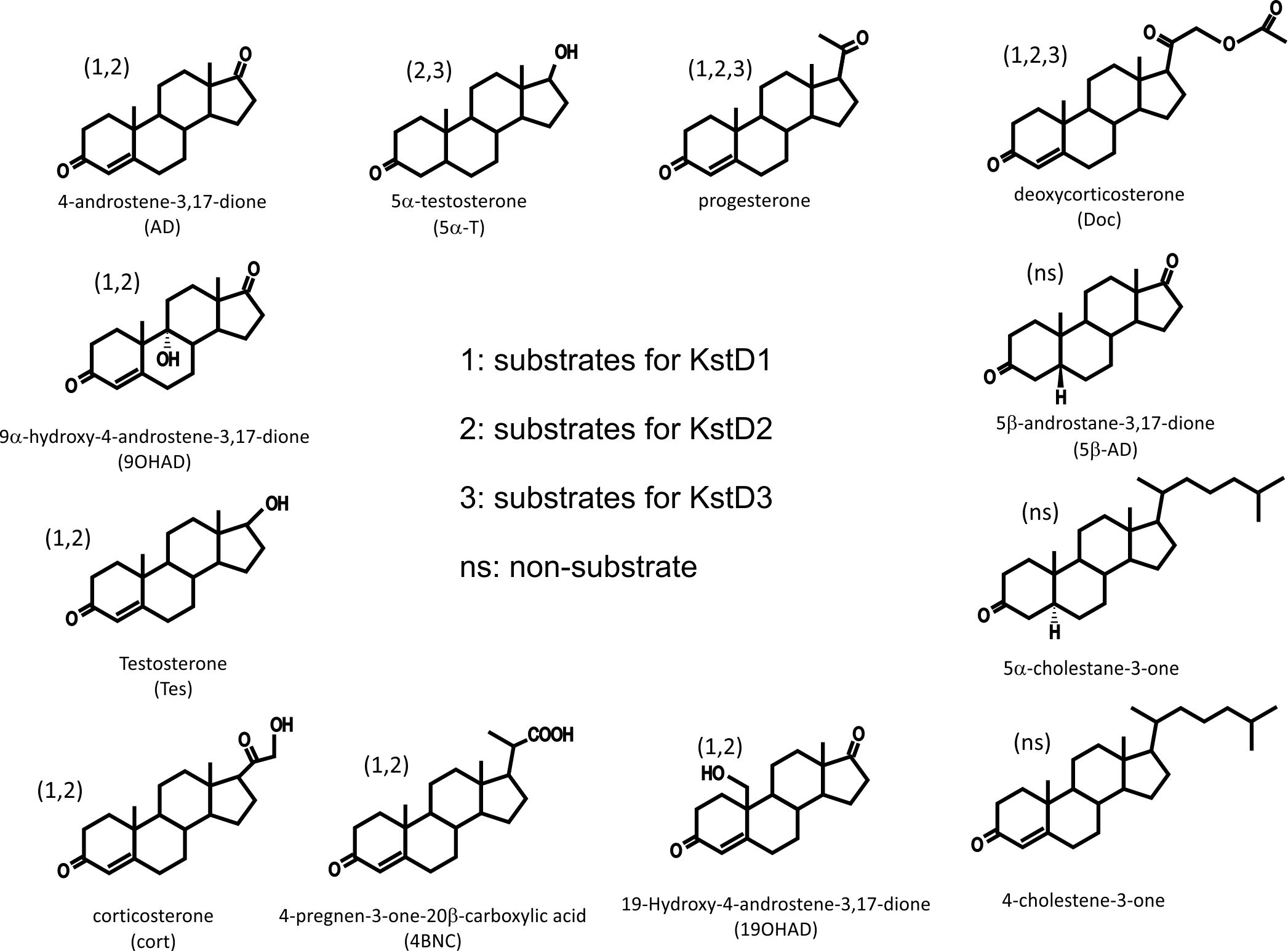

Supplement: Supplementary file 3 — Additional file 3. Structure of the steroids used in this work. [file 12934_2017_657_MOESM3_ESM.jpg]

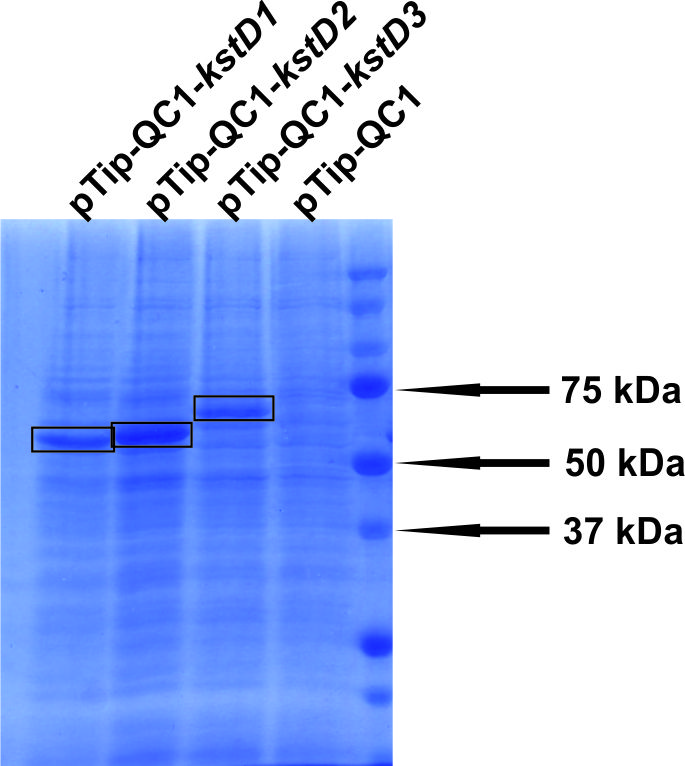

Supplement: Supplementary file 5 — Additional file 5. Expression of KstDs from ptip-QC1 vectors in induced R. erythropolis CECT3014 cells. SDS-PAGE analysis on a 12.5% gel was performed using 10 µg of the cell-free extracts. The band corresponding to KstD overexpression is marked with a rectangle. Precision plus protein standard from Bio-Rad was used as size marker. [file 12934_2017_657_MOESM5_ESM.jpg]
