# Supplementary material for: Functional differentiation of 3-ketosteroid Δ1-dehydrogenase isozymes in Rhodococcus ruber strain Chol-4
Source: Microb Cell Fact. 2017 Mar 14;16:42. doi: 10.1186/s12934-017-0657-1 (PMC5348764; doi:10.1186/s12934-017-0657-1)
Supplement: Supplementary file 4 — Additional file 4. Scheme of the pNVSP vectors construction. The shuttle vector pNV119 was modified to include a mcs from pSEVA351 (pNVS) and after that coupled with the apramycin resistance ORF. Intergenic regions containing the putative kstD promoters were cloned in the mcs to obtain pNVSP vectors. [file 12934_2017_657_MOESM4_ESM.pptx]

## Slide 1
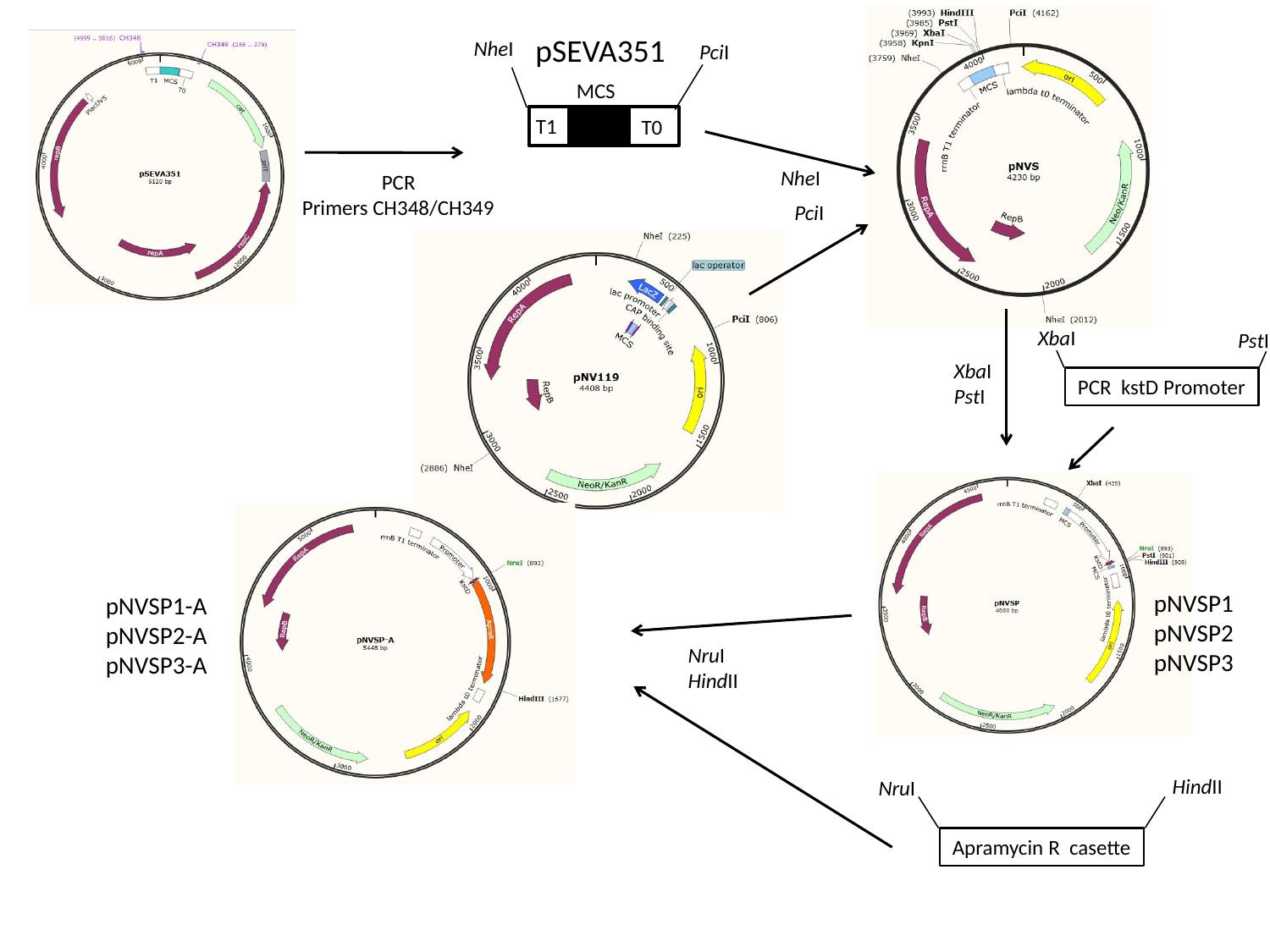

pSEVA351
NheI
PciI
MCS
T1
T0
NheI
PCR
Primers CH348/CH349
PciI
XbaI
PstI
XbaI
PstI
PCR kstD Promoter
pNVSP1
pNVSP2
pNVSP3
pNVSP1-A
pNVSP2-A
pNVSP3-A
NruI
HindII
HindII
NruI
Apramycin R casette
