# Supplementary material for: Functional differentiation of 3-ketosteroid Δ1-dehydrogenase isozymes in Rhodococcus ruber strain Chol-4
Source: Microb Cell Fact. 2017 Mar 14;16:42. doi: 10.1186/s12934-017-0657-1 (PMC5348764; doi:10.1186/s12934-017-0657-1)
Supplement: Supplementary file 6 — Additional file 6. R. ruber strain Chol-4 ORF4 and ORF5 protein sequences. [file 12934_2017_657_MOESM6_ESM.docx]

**Additional file 6.** *R. ruber* strain Chol-4 ORF4 and ORF5 protein sequences.

ORF4 (FAD-binding dehydrogenase)

MNPATVSESLTYDVVVVGSGAGGLSAAVAAAHGGASVLVVEKADVCGGATAWSGGWMWTPRNLFAHADGVHEDRAQPRRYLEHRLGDEFDAAKVDAFLDGAPEMVEFFERHTALQFVPGAAIADIHGDTPGAGTGHRSVAPKPVSLRRLGSDVAALLRRQLYETSFLGMGIMAGPDLQAFLHSTRSGRAFVHCARRVSTHMLDLATERRGQQLVNGTALVGRLLRSALDAGVDIRVATAATALVTDPSGRVTGVRIDGPGGARTVSARRGVVLATGGFTHDIDRRRELFPRTPSGREHWTLTPPTTTGDGISLGESVGGRLDRSLASPVAYCPVSLVRYRNGRQGVFPHILDRGKPGVIGVLADGRRFVNEALGYHDYTLAMIEQVPDGEEVCSWLIADQQYLRYFPLGMAKPFPIPTWPYLRSGYLSKGRTIRDLAEKIGVDPDGLEKTVTAFNESARVGEDPEFGRGTTPFNVKSGDADNPWPNPSLAPLERGPFYAVKVVPGSFGTFAGLVTDSSSRVLNGDDRPIDGLFAVGVDQSSVMGGHYPSGGINLGPAMTFGYLTGRRLASTTGATR

ORF5 (Fumarate reductase)

MQHNEEAAEYDVVVLGSGAAGLCAALSAARSGARVGVFEKGELLGGTTCLSSAVAWLPNNRYAREAGIADSREGALAYLESLSHGMILPELAEAFVDTVPELLEWLDTTPLKMRLVAGYPDYHPERPGGMPHGGRSLEPELFSFIDLGRWEDKLVGVPRRMTVTETPIGGGTGHLPADVQEQRERDHVEGLGRGMVAALLQGCLDEGVAVHTGERGVRLIQDESGRVTGVRFEGRGGPHDVLAEHGVVLATGGFEWDERLRRDFLRGPLAHPATVPTSTGDGLRMAMRVGAQLGNMREAWWAPVAVLPGQRANGAQAVQLVHRERTAPHSIMVNRHGRRFTNEATNYNALGGAFHHLDAHDFDYPNQPCWLIFDADHVEKYGAFGAAPGTEAPEWVVRADTLADLARQIEVPAAALESTVAHWNEDVRRGHDSEYHRGESVYDGFVGDKNKYPGVESTLGPVARAPFHAVQIHSSTLGTKGGPRTDADGAVLDVDDRVIPGLFAAGNVMAAPTGMVYGGAGGTLGPALVFGYRAGRAAARAAGSPSEPGGVLREPTTV
